# Socioecological Factors Associated with Hypertension Awareness and Control Among Older Adults in Brazil and Colombia: Correlational Analysis from the International Mobility in Aging Study

**DOI:** 10.5334/gh.1282

**Published:** 2023-12-26

**Authors:** Catherine M. Pirkle, Ricardo Oliveira Guerra, Fernando Gómez, Emmanuelle Belanger, Tetine Sentell

**Affiliations:** 1 Office of Public Health Studies, University of Hawaiʻi at Mānoa, 1960 East-West Road, BioMed T102A, Honolulu, HI 96822-2319, US; 2 Universidade Federal do Rio Grande do Norte, Departamento de Fisioterapia, Campus Universitário –Av. Salgado Filho S/N. 59078 970, Natal-RN Brasil, BR; 3 Research Group on Geriatrics and Gerontology, Faculty of Health Sciences, Universidad de Caldas, Manizales, CO; 4 Sede Principal Calle 65 No 26; 5 Department of Health Services, Policy & Practice, Center for Gerontology and Healthcare Research, Brown University School of Public Health, 121 South Main Street, 6^th^Floor, Providence, RI, US; 6 Office of Public Health Studies, University of Hawaiʻi at Mānoa, 1960 East-West Road, BioMed D209E, Honolulu, HI 96822-2319, US

**Keywords:** hypertension, older adults, socioecological factors, Latin America

## Abstract

**Background:**

Hypertension awareness and control are understudied among older adults in middle-income countries, with limited work contextualizing awareness and control across layers of influence (individual to the community). Research on hypertension in Latin America is acknowledged as insufficient.

**Objectives:**

This study applies the socioecological model (SEM) to examine individual, interpersonal, institutional, and community factors related to hypertension awareness and control in older adults residing in Brazil and Colombia. It identifies groups of older adults more likely to be unaware of their condition and/or to have challenges achieving hypertension control.

**Methods:**

We analyzed International Mobility in Aging Study data of 803 community-dwelling adults 65–74 years from study sites in the two most populous countries in South America. The study framework was the socioecological model. Logistic regression models identified factors associated with hypertension awareness and control.

**Conclusions:**

Hypertension was prevalent in both samples (>70%), and awareness was high (>80%). Blood pressure control among diagnosed respondents was low: 30% in Brazil and 51% in Colombia. Factors across the socioecological model were associated with awareness and control, with notable differences across countries. Those with diabetes (OR 4.19, 95%CI 1.64–10.71) and insufficient incomes (OR: 1.85, 95%CI 1.03–3.31) were more likely to be aware of their hypertension. In Colombia, those reporting no community activity engagement were less likely to be aware compared to those reporting community activities. In Brazil, it was the opposite. Women (OR 1.66, 95%CI 1.12–2.46) and those reporting strolling shops and stores (OR 1.80, 95% CI 1.09–3.00) were significantly more likely to have their hypertension under control. In Brazil, those 70–75 were significantly less likely to have their hypertension under control compared to their younger counterparts. In Colombia, this was not observed. This paper highlights the importance of theory-based studies within unique Latin American contexts on hypertension and suggests novel opportunities for intervention.

## Introduction

Cardiovascular disease (CVD) accounts for almost a third of all deaths globally [1] including in the Latin America and Caribbean region (LAC) [2]. Hypertension, or high blood pressure, describes a physiological state in which the force of blood flowing through blood vessels is too high, which can damage tissues inside the arteries and narrow them [3]. Hypertension is a critical risk factor for CVD and it is the leading cause of deaths from heart attacks and stroke [4]. Hypertension studies warrant global attention because it is the strongest modifiable contributor to the global burden of disease and global mortality [5]. According to the World Health Organization (WHO), over a billion people currently live with hypertension [4]. In the LAC region, adult hypertension prevalence is estimated between 20% to 40% [2].

Hypertension prevalence increases with chronological age; therefore, proper management in older adults is of particular importance [5]. In LACs, it is estimated 16% of men and 13% of women ages 20–29 have hypertension, while 28% of men and 34% of women ages 40 to 49 have the condition. By age 70, hypertension prevalence estimates are 60% and 74% for men and women, respectively [6], highlighting its ubiquity in this older group and the need for proper management.

Hypertension control among older adults confers substantial clinical and public health benefits [7]. When an individual is diagnosed, the target is to bring his or her high blood pressure below 140/90 mmHg to achieve control [2]. There are multiple methods to control hypertension including smoking cessation, nutritious diet, regular physical activity, and medication [7,8,9]. Control is predicated on awareness, which requires access to a health professional, appropriate diagnostics and effective provider-patient communication [9,10,11]. Studies on the determinants of hypertension control and awareness are insufficient across most of Latin America, highlighting an important research gap [2]. Work specifically focused on these contexts is needed given recent and rapid changes in socioeconomic development, including a widening of social inequalities and profound modifications in health care policies and practices [12,13,14].

Hypertension awareness and control appear generally understudied among older adults in middle-income countries, including the LAC region, particularly when contextualized according to interpersonal, institutional, and community factors. Research on hypertension is usually limited to individual demographics and/or behaviors [8,15,16]. However, a rich body of evidence demonstrates an individual’s response to and management of a condition, such as hypertension, can vary due to intrapersonal, interpersonal, sociocultural, and economic factors [17,18,19]. Many of these factors may influence hypertension management and examination of these factors may provide novel insights into facilitators of and barriers to hypertension awareness and control.

This study applies the socioecological model (SEM) to examine individual, interpersonal, institutional, and community factors related to hypertension awareness and control in older adults residing in two Latin American countries. In Brazil, the most populous country of South America, hypertension is the second most important risk factor of disability and death [20]. In Colombia, the second most populous country in South America, hypertension constitutes the primary cause of disability and death [21].

## Methods

### Design and Participants

This is a cross-sectional analysis of International Mobility in Aging Study (IMIAS) 2012 baseline data of community-dwelling adults 65–74 years of age from two study sites: Natal, Brazil (n = 402) and Manizales, Colombia (n = 407). A detailed description of the IMIAS is available elsewhere [22,23].

### Setting and Procedures

Briefly, IMIAS recruited equal numbers of men and women registered at neighborhood health centers. The Natal sample was randomly drawn from the registers of the Family Health Program at three low-income and two middle-income areas of the city. Brazil has a public healthcare system (Sistema Único de Saúde or SUS) [24] and the Family Health Program (Programa Saúde da Família) is one of the strategies within the SUS to expand access to primary care to low- and middle-income communities. The Manizales sample was randomly drawn from the Public Health Insurance database. Colombia has the General Health and Social Security System which provides coverage to the population through contributory (social security system) and subsidized (public system) programs; in 2007, 82% of older adults were covered [25]. Information about the participants of these schemes is registered in the Public Health Insurance database. The acceptance rate was over 95% at both Latin American sites. Respondents at both Latin American sites are representative of older adults from the cities from which the samples originated [22].

Those with severe cognitive impairment, determined by four or more errors on the orientation scale of the Leganes Cognitive Test, were excluded to ensure study procedures could ethically be completed. Two people in Manizales and five people in Natal were excluded [22].

Survey questionnaires were administered at respondents’ homes by trained interviewers. Additional procedures included checking the respondents’ blood pressure and anthropometric measures. Respondents were asked to bring the containers of their current medications. The interviewer recorded medication names and a trained pharmacist coded the medications according to their approved uses. All study instruments were translated into the appropriate language (Portuguese, Spanish) and interviews were conducted in the language of the site [22].

The research ethics committees of the Universidad de Caldas and the Universidade Federal do Rio Grande do Norte approved this study and written informed consent was obtained by all participants prior to participating in the study.

### Conceptual Framework

The SEM applies a broad perspective to understand multiple factors affecting health outcomes [26]. Accordingly, individual-level health outcomes, knowledge, and behaviors are influenced by interpersonal (e.g., family, friends), organizational (e.g., health system), community (e.g., participation and engagement), and policy levels. The SEM is commonly used for studies of health outcomes, especially lifestyle risk factors for chronic disease [27,28,29]. This model is relevant to this work as research from diverse locations including Europe, Asia, Africa, and Latin America have shown social and environmental factors are strong determinants of health in older adults [30,31,32,33].

### Outcome Measure: Hypertension Awareness and Control

Blood pressure was measured three times in succession, one minute apart, on the same arm, with the respondent in a sitting position. A validated automated blood pressure device—Omron M3 (Omron Corp., Kyoto Japan)—was used in both samples after the respondent sat at rest for at least five minutes. The mean value of the second and third measurements was calculated as the respondent’s blood pressure. Clinical hypertension was defined by a measured systolic blood pressure of ≥140 and/or diastolic blood pressure of ≥90 mmHg [34].

Self-reported, health professional-diagnosed hypertension was also recorded based on an affirmative response to the question, ‘Has a doctor or nurse ever told you that you have high blood pressure or hypertension?’ Self-reported hypertension has been previously validated and tends to be highly specific (e.g., low probability that non-hypertensive persons are classified as hypertensive) [35,36].

Using the measured blood pressure values and the self-reported diagnosis variable, three outcome measures were created: (i) hypertension y/n, which included those detected through blood pressure measurement and/or who reported a clinical diagnosis; (ii) hypertension awareness y/n, which encompassed only those reporting doctor diagnosed hypertension among those with hypertension; and (iii) hypertension control y/n, defined as reporting a hypertension diagnosis and having a measured blood pressure <140/90, among those reporting a hypertension diagnosis.

### Risk Factors Informed by the Socioecological Model

Individual and behavioral: These included chronic conditions (diabetes, obesity), sociodemographic characteristics (sex/gender, education, income sufficiency), and health behaviors (smoking, alcohol consumption, exercise). Both diabetes and obesity are well-known risk factors of hypertension [37]. Respondents were coded as having diabetes if they responded affirmatively to the question, ‘Has a doctor or nurse ever told you that you have diabetes, that is to say, high blood sugar levels?’ or if they were taking medication for diabetes. Others have validated self-reported health professional diagnosis with diabetes [38]. Consistent with WHO guidelines, a BMI of 30 kg/m^2^ or greater was considered obese [39]. Age (in years) was self-reported. Sex/gender was recorded by the interviewer. Education was self-reported based on the question, ‘What is the highest level of schooling that you have completed?’ and dichotomized into secondary or post-secondary education versus primary only or illiterate. Income sufficiency was self-reported according to the question, ‘To what extent does your income allow you to meet your needs?’ Respondents were coded as having sufficient incomes if they responded ‘Very well’ or ‘Suitably’; otherwise, their income was categorized as insufficient. Respondents who reported that they currently or formerly smoked were categorized as smokers. Those who reported ever drinking alcohol were coded as consumers. Finally, the amount of respondents’ walking exercise was estimated using a validated computer animated assessment tool, the Mobility Assessment Tool for Walking—MAT-W. Walking is the most common type of moderate-intensity physical activity for older adults [40]. The MAT-W has been validated against the mCHAMPS5 self-report physical activity questionnaire and accelerometery [41]. The average minutes of walking physical activity was dichotomized into 30 min/day, yes or no.

Interpersonal: Social relationships and support impact hypertension control outcomes, including awareness and level of control [30,42,43]. The quality of respondents’ relationships was assessed with the validated IMIAS-Social Support Social Networks scale [44]. Support provided by relationships was assessed by: feeling loved and appreciated, listened to, importance of the respondent’s role in the relationship, usefulness to the other person, and overall satisfaction with the relationship. Support was ascribed according to types of social ties— partner, children, family, and friends [44]. Psychometric assessments were conducted to confirm the measurement validity of this scale for each type of social tie. Because of wide differences in the nature of social relationships across various IMIAS sites, responses were coded using site-specific cut-offs for no social tie, low social support (lowest site-specific quartile), and high social support (other three quartiles) across each kind of social tie [44]. Responses were dichotomized for ease of interpretation. Low social support, including no support, is the reference value used to estimate models.

Institutional: These questions relate to respondents’ access and utilization of health care services, assuming individuals accessing services are more likely to be diagnosed and controlled. Whether the respondent had a usual source of care was determined by the question, ‘Do you have a regular medical doctor or clinic for medical care? y/n.’ Another question was, ‘How many times have you gone to the doctor in the last year?’ Those reporting six or more visits per year were categorized as having frequent medical visits, as this was the upper quartile of reported visits per year. This study also recorded whether a respondent had taken medications in the past two weeks, y/n, based on the assumption medication use reflected access to health services. Because there was no variability in access to a usual source of care in Manizales (99% reported access), this variable was excluded in the multivariate analyses and only included in the descriptive analyses for comparison purposes with Natal.

Community and environment: Respondents were asked if they engaged in religious activities, attended community or recreational center(s), and/or were members of a professional association. If the respondent reported any such activities, this was coded as yes for that specific community activity. Respondents were also queried on their engagement in his/her environment—Do you stroll shops or stores?

### Statistical Analyses

Descriptive statistics were calculated for hypertension prevalence, awareness and control among respondents with valid observations for these measures (803 of the 809 IMIAS respondents from Brazil and Colombia). The sample was described according to variables in the SEM and compared with the chi-square test differences by country. These analyses were completed for respondents with valid observations for all factors of the SEM described in the measures section (n = 775).

In bivariate analyses, stratified by study site, hypertension awareness and control across factors from the SEM were compared with a chi-square test for independent proportions. To identify factors independently associated with awareness and control, multivariate logistic regression models were constructed. First, all factors with p-values of ≤0.20 from the bivariate analyses were included. When the bivariate analyses indicated possible differences in the direction of association between study site and a factor (e.g., education, high partners ties, and community activities for hypertension awareness and age group for hypertension control), an interaction term was introduced to the model between study site and the factor. Next, all factors in the preliminary model with a p-value >0.20 were dropped to obtain a more parsimonious model. Then, the dropped factors were added one by one to the parsimonious model to assess if their re-introduction changed any of the other coefficients (none did). All factors with a p-value of ≤0.10 in the final models were retained, except for sex and age, which were included irrespective of p-value based on theory. Finally, a model for goodness of fit and test for multicolinearity (tolerance and variance inflection factor) were conducted. Analyses were completed using STATA 13.0 (College Station, TX).

## Results

**Figure 1:**
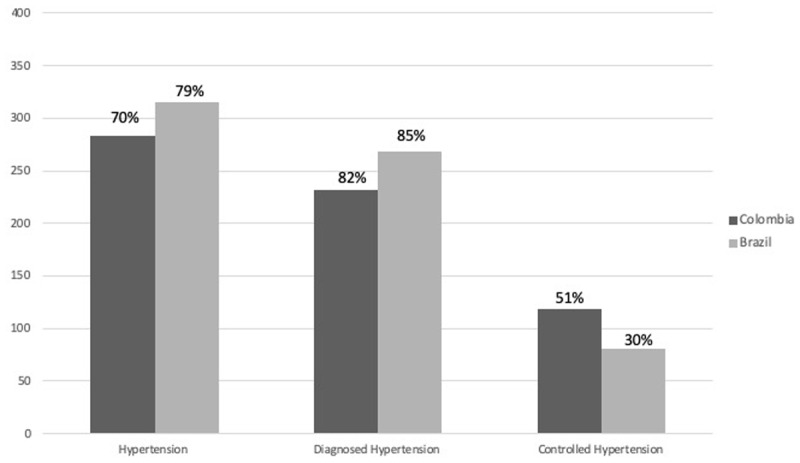
Number and percent of Brazilian (N = 400) and Colombian (N = 403) IMIAS respondents with detected or reported hypertension; number (%) of respondents with hypertension that reported a diagnosis (awareness); number (%) of diagnosed respondents who were under control.

Doctor-diagnosed hypertension was reported by 58% of Colombian and 67% of Brazilian respondents. Hypertension estimates increased to 70% of Colombian and 79% of Brazilian respondents when clinical hypertension, obtained from measured blood pressure, was included with the self-reported diagnoses. At each site, the proportion of hypertensive respondents who were aware of their condition was 82% and 85% in Colombia and Brazil, respectively. However, hypertension control among diagnosed respondents was only 51% in Colombia and 30% in Brazil. Figure 1 presents the cascade from hypertension detection, through awareness and control.

Table 1 presents the sample characteristics, by study site, according to individual, interpersonal, institutional, and community factors informed by the SEM. Compared to Colombia, respondents from Brazil were sicker and less educated. For example, nearly a third of Brazilian respondents reported a diabetes diagnosis, as compared to half that number of Colombian respondents. Brazilian respondents were also significantly more likely to report alcohol consumption, but less likely to report walking 30 minutes or more per day. For institutional characteristics, both samples showed evidence of high access to and use of health services, with nearly all Colombian respondents and two-thirds of Brazilian respondents reporting a usual source of care. Approximately 85% of respondents reported taking medications in the past two weeks, which also indicated access to health services. More Colombian respondents reported seeing a doctor six times or more a year than Brazilian respondents (31% versus 23%). Finally, with regard to the community variables, more Colombian respondents reported attending a community center and strolling shops and stores than Brazilian respondents.

**Table 1:** Factors Corresponding to Levels of the Socioecological (SE) Model among Latin American IMIAS respondents, by Study Site (n = 775).


LEVEL OF THE SE MODEL	VARIABLE NAME	MANIZALES, COLOMBIA (n = 382)	NATAL, BRAZIL (n = 393)	P VALUE FOR CHI2 (ACROSS)

**Individual-Level**				

**Comorbidities**	**Diabetes** ^1^			

	Yes	53 (13.9%)	125 (31.8%)	<0.01

	**Obesity** ^2^			

	Yes	65 (17.0%)	106 (27.0%)	<0.01

**Sociodemographics**				

	**Age**			

	70–75 years	178 (46.6%)	183 (46.6%)	0.99

	**Gender**			

	Female	190 (49.7%)	208 (52.9%)	0.38

	**Education**			

	Secondary or post-secondary	63 (16.5%)	41 (10.4%)	0.01

	**Perceived income insufficiency**			

	Yes	269 (70.4%)	291 (74.1%)	0.26

**Behavioral**				

	**Smoke** ^3^			

	Yes	201(52.6%)	198 (50.4%)	0.53

	**Alcohol** ^4^			

	Yes	240 (62.8%)	325 (82.7%)	<0.01

	**Walking, 30 min/day** ^5^			

	Yes	131 (34.3%)	87 (22.1%)	<0.01

**Interpersonal**				

	**Social ties: partner** ^6^			

	High	139 (36.4%)	173 (44.0%)	0.03

	**Social ties: children**			

	High	239 (62.6%)	272 (69.2%)	0.05

	**Social ties: family**			

	High	282 (73.8%)	286 (72.8%)	0.74

	**Social ties: friends**			

	High	210 (55.0%)	144 (36.6%)	<0.01

**Institutional**				

	**Usual Source of Care**			

	Yes	378 (99.0%)	252 (64.1%)	<0.01

	**Frequent visits to doctor** ^7^			

	Yes	117 (30.6%)	90 (22.9%)	0.02

	**Medication taken in past 2 weeks** ^8^			

	Yes	319 (83.5%)	334 (85.0%)	0.57

**Community**				

	**Participate in Religious Activities**			

	Yes	348 (91.1%)	357 (90.8%)	0.90

	**Attend community center**			

	Yes	137 (35.9%)	100 (25.5%)	<0.01

	**Member of professional association**			

	Yes	114 (29.8%)	125 (31.8%)	0.55

	**Stroll shops/stores**			

	Yes	317 (83.0%)	303 (77.1%)	0.04


^1^ Self-reported doctor diagnosed diabetes or taking diabetes medication (all medications were shown to the interviewer and recorded). ^2^ Defined as a Body Mass Index (BMI) of 30 or greater. ^3^ This is defined by self-report. Current and former smokers are categorized as yes, while never smokers are categorized as no. ^4^ This is defined according to self-report. Those who report ever drinking alcohol are coded as yes, all others as no. ^5^ This is defined using a validated computer animated assessment tool that asked about usual, leisure, and fast walking. ^6^ All social ties measures compare high to low or none. ^7^ 6 or more reported visits to the doctor per year. ^8^ Self-report of taking medication in the past 2 weeks.

### Hypertension Awareness

While hypertension awareness was relatively high in both samples, there were a number of factors across multiple levels of the SEM associated with awareness. Bivariate associations between factors informed by the SEM and hypertension awareness are presented in Table 2. In both countries, hypertension awareness was significantly higher among respondents with diabetes, women, those with frequent medical visits, and those reporting taking medication within the last two weeks. There were several factors significantly associated with higher hypertension awareness, in either country: obesity, having never smoked, high level of social support from one’s domestic partner, engagement in community activities, and engagement in professional activities. In Brazil, those with higher levels of support from their partner were significantly less likely to be aware of their hypertension compared to those with low support. While not significant, in Colombia, the direction of the association was the opposite. This difference in the direction of association between countries was also observed for engagement in community activities.

**Table 2:** Bivariate associations between factors corresponding to the socio-ecological model and hypertension awareness and control, by study site (N = 575).


	MANIZALES						NATAL					
	
	n	AWARE	p-VALUE	n	CONTROL	p-VALUE	n	AWARE	p-VALUE	n	CONTROL	p-VALUE

Diabetes	42	41 (97.6%)	<0.01	41	19 (46.3%)	0.54	106	101 (95.3%)	<0.01	101	25 (24.8%)	0.12

Obesity	53	47 (88.7%)	0.12	47	24 (51.1%)	0.95	94	88 (93.6%)	0.01	88	21 (23.9%)	0.11

70–75 years	122	101 (82.8%)	0.56	101	53 (52.5%)	0.62	147	126 (85.7%)	1.00	126	30 (23.8%)	0.03

Female	135	117 (86.7%)	0.02	117	66 (56.4%)	0.07	168	155 (92.3%)	<0.01	155	52 (33.6%)	0.17

(Post) secondary	42	31 (73.8%)	0.18	31	17 (54.8%)	0.62	30	26 (86.7%)	0.88	26	7 (26.9%)	0.69

Income insufficiency	190	156 (82.1%)	0.58	156	77 (49.4%)	0.53	230	202 (87.8%)	0.07	202	59 (29.2%)	0.49

Ever smoke	137	104 (75.9%)	0.02	104	48 (46.2%)	0.20	154	129 (83.8%)	0.33	129	32 (24.8%)	0.06

Ever alcohol	174	147 (84.5%)	0.07	147	72 (49.0%)	0.47	255	221 (86.7%)	0.30	221	68 (30.8%)	0.71

30 min/day exercise	91	71 (78.0%)	0.33	71	40 (56.3%)	0.25	64	54 (84.4%)	0.73	54	17 (31.5%)	0.83

High social support from partner	105	87 (82.9%)	0.60	87	47 (54.0%)	0.42	127	103 (81.1%)	0.05	103	29 (28.2%)	0.54

High social support from children	171	141 (82.5%)	0.51	141	73 (51.8%)	0.66	209	180 (86.1%)	0.77	180	58 (32.2%)	0.32

High social support from family	201	167 (83.1%)	0.19	167	83 (49.7%)	0.59	221	190 (86.0%)	0.84	190	57 (30.0%)	0.86

High social support from friends	147	123 (83.7%)	0.27	123	58 (47.2%)	0.23	106	94 (88.7%)	0.28	94	28 (29.8%)	0.89

Usual source care	264	215 (81.4%)	0.51	215	110 (51.2%)	0.15	201	180 (90.0%)	<0.01	180	62 (34.4%)	0.03

Frequent MD visits	87	81 (93.1%)	<0.01	81	42 (51.9%)	0.79	74	71 (96.0%)	<0.01	71	27 (38.0%)	0.10

Medication last 2 weeks	239	210 (87.9%)	<0.01	210	109 (51.9%)	0.05	276	252 (91.3%)	<0.01	252	79 (31.4%)	0.10

Religious activities	242	197 (81.4%)	0.86	197	104 (52.8%)	0.05	282	243 (86.2%)	0.45	243	73 (30.0%)	0.75

Community activities	96	86 (89.6%)	0.01	86	48 (55.8%)	0.22	82	66 (80.5%)	0.11	66	22 (33.3%)	0.54

Prof. association	80	73 (91.3%)	0.01	144	76 (52.8%)	0.39	93	81 (87.1%)	0.65	183	49 (26.8%)	0.61

Stroll shops	220	180 (81.8%)	0.622	180	94 (52.2%)	0.32	237	204 (86.1%)	0.74	204	69 (33.8%)	0.02

**Total**	**217**	**81.3%**	**NA**	**110**	**50.7%**	**NA**	**264**	**85.7%**	**NA**	**80**	**30.3%**	**NA**


Table 3 presents the multivariate associations for hypertension awareness. The strongest associations were observed for taking medication in the last month and for diabetes. Income insufficiency was also statistically significantly associated with hypertension awareness. There were also statistically marginal associations for obesity (OR 1.98, 95%CI 0.94–4.14) and secondary or post-secondary education (OR 0.53, 95%CI 0.25–1.11). Finally, there was a statistically significant interaction between study site and engagement in community activities. In Colombia, those reporting no community activity engagement were less likely to be aware of their condition compared to those reporting community activities. In contrast, in Brazil, it was the opposite. Those reporting community activity engagement were less likely to be aware of their hypertension.

**Table 3:** Factors associated with hypertension awareness among Brazilian and Colombian IMIAS respondents (n = 575).


	ODDS RATIO	95% CI	P-VALUE

Diabetes	4.19	1.64–10.71	<0.01

Obese	1.98	0.94–4.14	0.07

70–75 years	0.92	0.54–1.57	0.75

Female	1.50	0.84–2.68	0.17

(Post)secondary education	0.53	0.25–1.11	0.09

Income insufficiency	1.85	1.03–3.31	0.04

Medication taken in last 2 weeks	18.86	9.61–37.41	<0.01

Site & Community activities interaction			

Colombia, no community activities	0.56	0.29–1.06	0.08

Colombia, community activities	1.16	0.47–2.86	0.74

Brazil, community activities	0.42	0.18–0.95	0.04


Odds ratios obtained from logistic regression model.

### Hypertension Control

Table 2 presents the bivariate results between factors informed by the SEM and hypertension control by study site. There were no statistically significant factors in both sites, but the directions of the associations were generally the same for factors that were significant or marginally significant in one site, but not the other. For example, women at both sites were slightly more likely to have controlled blood pressure than their male counterparts, as were those who reported strolling shops and stores compared to those who did not. In contrast, Brazilian respondents aged 70–75 were significantly less likely to have controlled blood pressure compared to younger respondents from this site. This relationship was not observed in Colombia.

Table 4 presents the multivariate analyses for hypertension control. Women and those reporting strolling shops and stores were significantly more likely to have their hypertension under control. There were marginal associations for those reporting frequent medical visits (OR 1.43, 95%CI 0.94–2.16) and diabetes (OR 0.65, 95%CI 0.42–1.02). There was also a statistically significant interaction between study site and age group. Brazilian respondents were less likely to be under control than Colombian ones. In Colombia, there was no significant difference in the likelihood of hypertension control by age group. However, in Brazil, those 70–75 were significantly less likely to have their hypertension under control compared to their younger counterparts, especially when compared to Colombian respondents of both age groups.

**Table 4:** Factors associated with hypertension control among Brazilian and Colombian IMIAS respondents (n = 481).


	ODDS RATIO	95% CI	P-VALUE

Female	1.66	1.12–2.46	0.01

Diabetes	0.65	0.42–1.02	0.06

Frequent MD visits	1.43	0.94–2.16	0.09

Stroll shops	1.80	1.09–3.00	0.02

Site & Age Interaction			

Colombia, 64–69	1.63	0.97–2.73	0.07

Colombia, 70–75	1.75	1.02–3.00	0.04

Brazil, 70–75	0.57	0.33–0.99	0.05


Odds ratios obtained from logistic regression model.

## Discussion

In this sample of community-dwelling older adults, the SEM was applied to examine individual, interpersonal, institutional, and community factors related to hypertension awareness and control in Brazil and Colombia. While individual-level factors such as diabetes were associated with both hypertension awareness and control, associations were also observed with various institutional- and community-level factors. A notable finding was the significant country differences in factors associated with awareness and control. In Colombia, engagement in community activities was associated with higher hypertension awareness. The opposite was observed in Brazil. Similarly, there was no difference in hypertension awareness by age group in Colombia, while in Brazil, the older group (70–75 years) was more poorly controlled than the 64–69 group. This study contributes to the literature by highlighting the importance of research within unique contexts [30]; others conducting cross-cultural work have also observed surprising variations in predictors of health outcomes across middle-income contexts [45,46].

Hypertension was prevalent in both country samples, with approximately three quarters of Brazilian and Colombian respondents either reporting a diagnosis or having a mean blood pressure of ≥140/90, taken as part of the study procedures. Prevalence estimates in this study were comparable to those of other low- and middle-income countries based on the WHO’s Study of Global Ageing and Adult Health (SAGE). In this study of over 35,000 adults 50 years and older from China, Ghana, India, Mexico, the Russian Federation and South Africa, 53% (range 32% in India to 78% in South Africa) had hypertension [46]. Similarly, in a national study of over 20,000 older Colombians, 58% (95%CI 55–50) had hypertension [47], while a national study of nearly 12,000 Brazilian adults 60 years and older reported a prevalence of hypertension of 67% [48]. Finally, a study of urban adults from Midwest Brazil, with data collection occurring just before IMIAS baseline data were collected, reported a prevalence of hypertension of 75%, which is very similar to results from this study [49]. Since hypertension is a modifiable risk factor for most common causes of morbidity and mortality in older adults, these results highlight a need to improve the detection, treatment and control of hypertension among older adults in the LAC region [2,11,50].

Among respondents with hypertension, awareness was high (>80%) in both countries; awareness was slightly higher among Brazilian than Colombian respondents. This finding contrasts with the SAGE study. With the exception of the Russian Federation (72%), no more than 45% of respondents were aware of their hypertension in the other five countries [46]. The WHO Region of the Americas, which includes Brazil and Colombia, has ambitious goals for the control of hypertension, including achieving an awareness level of 70% or more [2]. Here, hypertension awareness was higher than the 70% target. It was also higher than levels reported in the Cardiovascular Risk Factor Multiple Evaluation in Latin America (CARMELA) trial and the Perspective Urban Rural Epidemiological (PURE) study for South America [2]. In CARMELA, which included respondents younger than the samples in this study, 31% of Bogotá respondents were unaware of their hypertension and a similar proportion of those with hypertension were controlled [50]. In contrast, 2015 data from the Healthcare, Welfare, and Ageing Survey (SABE), which is a population survey of older adults in Colombia, reports an identical percent of hypertension awareness (82%) as was observed among Colombian participants in this study [51]. Blood pressure control among respondents aware of their condition in this study was low, especially in Brazil, where less than a third of respondents achieved control. Similar to this study and CARMELA [50], SAGE also reported very low levels of control among those aware of their hypertension [46].

Hypertension awareness was strongly associated with use of health services, specifically frequent medical visits and taking medication. The high awareness observed among Brazilian and Colombian respondents is consistent with the elevated levels of access to health services reported by them. In Colombia, 99% of respondents reported access to a usual source of care. The very high number of respondents in Colombia reporting a usual source of care is unsurprising given the high insurance coverage of older adults in the country. Results from the SABE study of older adults in Colombia reported 98% coverage with any type of health insurance [52]. In Brazil, about two-thirds of respondents reported access to a usual source of care. Results from data collected between 2019–2021 by the Brazilian Longitudinal Study of Aging, which is a nationally representative population-based cohort study, reported 75% of older adults had any doctor visit in the past year [53]. The value is fairly consistent with the IMIAS findings on access to a usual source of care, especially considering that the state of Rio Grande do Norte, where Natal is located, is poorer than most other parts of the country. Individuals who use health care services, especially those taking a medication or frequently visiting a provider, are doing so for a reason. Thus, strong associations between measures of health care utilization and hypertension awareness are not surprising, especially after adjusting for factors associated with health care utilization patterns such as sex, education and income. Others working with LAC populations have likewise reported strong associations between accessing care and hypertension awareness [10,48].

There was a strong independent association observed between diabetes and hypertension. Hypertension and diabetes commonly occur together [54]. Those who use health services regularly, especially if they have a comorbid condition like diabetes or obesity, are likely to get diagnosed with the comorbidity of hypertension. This would explain the very high proportion (>95%) of individuals with diabetes who were aware of their hypertension status. Similar findings have been reported by others working in the LAC region [11]. The general relationship between chronic conditions and poor health, health service utilization, and hypertension awareness likely explains the finding that those with income sufficiency and higher educations were less aware of their hypertension. Older adults with lower incomes and educational attainment are more likely to be ill at older ages [22] and thus in contact with the health system.

In a cross-sectional study of hypertension correlates, it is expected that individuals with chronic conditions and those using health services would be aware of their hypertension status. Unexpectedly, there was an interaction between study site and community engagement on awareness. Brazilian respondents reporting engagement in community activities were less likely to be aware of their condition than those who did not. The opposite was observed in Colombia. One explanation is those who are busy engaging in community activities appear and feel healthy. Thus, they may consult with health care providers less frequently or providers perceive them as more robust than they actually are. While possible, it does not explain the site-specificity of the finding, which highlights the importance of considering contextual differences between communities. Previous research reports that topics discussed within social networks influence diagnosis and control of hypertension. Individuals may benefit from one another’s advice and experiences regarding disease management, but if the group is unlikely to communicate about health, the risk for undiagnosed hypertension actually increases [42]. The site-specificity of this study’s findings may reflect the nature and content of the community groups frequented at each site, including common topics of conversation.

This study also examined hypertension control and its correlates. Low levels of hypertension control have consistently been reported in Latin America [2,11,55,56]. Notably, control was much lower in Brazil than Colombia, which is consistent with fewer Brazilian respondents reporting a usual source of care. This observation is corroborated by findings from a 2013 national survey of over 60,000 Brazilian adults which reported a significant association between access to care with both awareness and control. Critically, this large study also highlighted variations in the content of care provided to those with diagnosed hypertension, as well as uneven quality [11]. Continuity of care is important for hypertension control; insufficient and/or inconsistent access to health services may critically interrupt treatment and management protocols, thereby resulting in poor control among those aware of their hypertension.

Women were significantly more likely than men to have controlled hypertension. Multiple studies report better hypertension control among women than men [8,10,11,49]. This result may be mediated by better medication adherence, as women tend to be more adherent than men in some contexts [57,58], but not all [59]. Women also tend to be more proactive about accessing and using health services [60].

Similar to the results for hypertension awareness, a community-level variable was associated with control—strolling shops and stores. Other research highlights the importance of social context to disease management and recognizes that much of the day-to-day work required to control hypertension occurs outside of the health care sector [42]. Strolling shops and stores may capture exercise the respondents did not consider in this study’s walking tool or may represent an enjoyable, blood-pressure lowering activity enjoyed by older Latin American adults. It may also capture facets of community engagement and social resources that other variables did not. Irrespective, it is an intriguing finding consistent with other research highlighting the importance of social and community resources for hypertension management [42].

A critical determinant of hypertension control is medication adherence among patients. The vast majority (>85%) of study respondents reported taking medication in the past two weeks, 70% of whom were taking antihypertensives. And yet, blood pressure control was low. This may point to patient adherence issues. Despite the effectiveness of antihypertensive medications, adherence is a known challenge and half of the patients prescribed an antihypertensive drug stop taking it within one year [61]. Low control also suggests health system and provider deficiencies, such as failure to apply evidence-based clinical guidelines or prescription of less effective drugs [61]. Given generally high rates of medication usage and high access to care (~100% in Colombia and 64% in Brazil), patient adherence issues alone are unlikely to explain the low overall control levels reported in this study and may point to a need to standardize treatment protocols, ensure access to affordable and effective drugs, and improve service delivery [2]. These suggestions are reinforced by the observation that control was only marginally better among those with regular visits with their health care provider (although, given the cross-sectional study design, many of those with poor hypertension control may need to see their provider more often). The study findings have regional importance. Coverage of health services has expanded considerably for older adults in Latin America over the past two decades. Policy changes have increased equity in access to health services regionally, reducing economic barriers to regular care and increasing the opportunity for better detection and management of hypertension [62]. However, results from this study highlight the need to evaluate the quality of these services, when accessed, to improve hypertension control.

This study has many strengths. A contribution of this work is the application of the socioecological approach to assess factors other than demographic traits and behaviors, permitting exploration of how social context shapes diagnosis and management. It also has some limitations. First, the sample size in this study is modest and the numbers of individuals with certain outcomes (e.g., lack of hypertension awareness) were small. The sample size also limited our ability to examine potentially interesting and informative interactions such as between social support variables and sex, by country. Such analyses could be of interest in future work from better powered studies. Second, while the purpose of this study was solely to highlight correlates of hypertension awareness and control, it is critical to reiterate its cross-sectional nature and that our purpose is not to draw conclusions about cause and effect. Third, while self-reported measures of disease status are commonly used and validated, misclassification is still possible. This misclassification would most likely be non-differential and thus bias effect estimates towards the null. Finally, our sample contained older adults from two urban centers and while results can be generalized to the older adults of those cities, further generalization may not be appropriate.

## Conclusions

Results from this analysis highlight groups more likely to be unaware of their condition and/or more likely to have challenges achieving hypertension control, in order to guide health system and health promotion activities around hypertension. Hypertension is increasing globally, due in part to population aging, but also to the increased exposure to hypertension risk factors—excess salt intake, calories, alcohol, and tobacco—related to economic development [4,60]. Since most low- and middle-income countries have weaker health systems than their more affluent counterparts, they also contain greater numbers of people with undiagnosed and uncontrolled hypertension [4,5,60]. This study points to general solutions for countries in the LAC region to improve hypertension outcomes for older adults, including continuing to build access to health care to increase awareness and emphasizing social activities, including strolling in shops, that may support hypertension control as well as other desirable public health outcomes (e.g., exercise, social networks). It also supports the need for country-specific work, as many factors varied across the two countries.

## Data Accessibility Statement

STATA code and a corresponding dataset can be provided upon request to the corresponding author. The questionnaire, datasets, codebook and other supporting information related to this study are available in the IMIAS repository: http://www.imias.ufrn.br. Additional details about the IMIAS cohort and how to access source data and materials is provided in the Cohort Profile [22].

## Abbreviations

**CI:** Confidence Interval

**CVD:** Cardiovascular Disease

**IMIAS:** International Mobility in Aging Study

**LAC:** Latin American and Caribbean

**OR**: Odds Ratio

**SEM**: Socioecological Model

**WHO**: World Health Organization
